# Spectral Analysis

**Published:** 1870-02

**Authors:** 


*Spectral Analysis.*—This subject was most ably and scientifically handled by *Prof. E. Lloyd Howard*, in a lecture before the *Peabody Institute*, of Baltimore, delivered January 27th. As this method of philosophical investigation is attracting deserved attention, we will endeavor in a future article, to give an abstract of the more important features of the lecture.

